# Risk Assessment of Polycyclic Aromatic Hydrocarbons and Heterocyclic Aromatic Amines in Processed Meat, Cooked Meat and Fish-Based Products Using the Margin of Exposure Approach

**DOI:** 10.21315/mjms2024.31.2.11

**Published:** 2024-04-23

**Authors:** Nurin Irdina Ab-latif, Rozaini Abdullah, Syaliza Omar, Maimunah Sanny

**Affiliations:** 1 Department of Food Science, Faculty of Food Science and Technology, Universiti Putra Malaysia, Selangor, Malaysia; 2 Department of Environmental and Occupational Health, Faculty of Medicine and Health Science, Universiti Putra Malaysia, Selangor, Malaysia; 3 Faculty of Pharmacy, Universiti Sultan Zainal Abidin, Besut Campus, Terengganu, Malaysia; 4 Laboratory of Food Safety and Food Integrity, Institute of Tropical Agricultural and Food Security, Universiti Putra Malaysia, Selangor, Malaysia

**Keywords:** risk assessment, polycyclic aromatic hydrocarbons, heterocyclic amines, margin of exposure, Benchmark Dose lower confidence limit

## Abstract

**Background:**

The objective of this study is to assess the risk of exposure of polycyclic aromatic hydrocarbons (PAHs) and heterocyclic aromatic amines (HCAs) in meat and fish-based products marketed in Malaysia using the margin of exposure (MOE) approach.

**Methods:**

Benchmark Dose (BMD) software was used to model the BMD at a lower end of a one-sided 95% confidence interval with a 10% incremental risk (BMDL_10_) of PAHs and HCAs from different target organ toxicities. The MOEs of PAHs and HCAs in meat and fish-based products were determined by utilising the calculated BMDL_10_ values and estimated daily intake of meat and fish-based products from published data.

**Results:**

The calculated BMDL_10_ values of PAHs (i.e. benzo[a]pyrene [BaP] and fluoranthene [FA]) and HCAs (i.e. 2-amino-3,8,dimethylimidazo[4,5-f]quinoxaline [MeIQx] and 2-amino-1-methyl-6-phenylimidazo[4,5,6]pyridine [PhIP]) ranged from 19 mg/kg bw/day to 71,801 mg/kg bw/day. The MOE of BaP ranged from 41,895 to 71,801 and that of FA ranged from 19 to 1412. As for MeIQx and PhIP, their MOEs ranged from 6,322 to 7,652 and from 2,362 to 14,390, respectively.

**Conclusion:**

The MOEs of FA, MeIQx and PhIP were lower than 10,000, indicating a high concern for human health and therefore demanding effective risk management actions.

## Introduction

Meat and fish are subjected to various heat treatments such as roasting, grilling, barbecuing and frying (1). Thermal treatment of meat may generate some undesired compounds, such as food-borne toxicants, despite increasing palatability and reducing microbiological risks (2). During the heat processing stage, the Maillard reaction, thermal decompositions and lipid oxidation reactions are essential chemical transformations that generate the building blocks or precursors of potential toxicants from carbohydrates, amino acids and lipids (3–5). Heterocyclic aromatic amines (HCAs) and polycyclic aromatic hydrocarbons (PAHs) are the two thermally generated food toxicants significantly formed during the thermal treatment of meat at high temperatures (6, 7). HCAs, highly mutagenic and potentially carcinogenic by-products, form during Maillard browning reactions, specifically in muscle-rich foods (8–10). With accumulating evidence, the International Agency for Research on Cancer (IARC) (11) has classified 2-amino-3-methylimidazo[4,5-f]quinoline (IQ) as Group 2A (probable human carcinogens) and MeIQ, MeIQx, DiMeIQx and PhIP as Group 2B (possible human carcinogens).

Conversely, PAHs can be formed through the incomplete combustion or pyrolysis (burning) of organic components, including fat, protein and carbohydrates, at a temperature above 200 °C, especially above 400 °C (12, 13). PAHs can be generated in the smoke produced when lipids are dropped onto flames. Consequently, known sources of PAH contamination in thermally treated proteinaceous foods are deposited on the food surface (14). The IARC has classified benzo[a]pyrene (BaP) as a human carcinogen (Group 1) and therefore, exposure to PAHs is a significant health concern (15). Consumption of grilled red meat increases the risk of intestine, breast, bladder, prostate and pancreas cancers, as reported in various epidemiological studies (16).

Risk assessment involves identifying, analysing and characterising a food-related health risk. It estimates the likelihood and severity of an adverse health effect from exposure to a hazard (17). Human exposure studies demonstrated that the magnitude of BaP dietary exposures is 2 ng/day–500 ng/day, which supersedes inhalation exposure of 10 ng/day–50 ng/day (18). Globally, the estimated average intakes of PAHs range from 0.02 μg/person/day to 3.6 μg/person/day, and in countries like India, Nigeria and China, the estimated average intakes of PAHs are 11 μg/person/day, 6 μg/person/day and 3.56 μg/person/day, respectively (19). Jahurul et al. (20) analysed three high-molecular-weight PAHs, namely, fluoranthene (FA), benzo[b]fluoranthene and BaP, in 42 types of meat and fish-based products widely consumed by the Malaysian population. The researchers estimated that the mean dietary intake of the sum of three PAHs was 297.58 ng/day. Earlier, the same authors determined the concentration of six predominant HCAs in meat and fish-based products and reported that the mean dietary intake of HCAs was 553.7 ng/day (21). In addition, the margin of exposure (MOE) approach is used to consider possible safety concerns arising from the presence of toxicants in food that is both genotoxic and carcinogenic. Kirkland et al. (22) updated the recommended lists of genotoxic and non-genotoxic chemicals for the assessment of the performance of genotoxicity tests and stated that PAHs (especially BaP) and HCAs (especially IQ) are both genotoxic and carcinogenic. However, the risk assessment of PAHs and HCAs reported in literature (12, 20, 21) was conducted quantitatively. Benford et al. (23) suggested qualitative risk assessment using the MOE approach for genotoxic and carcinogenic substances such as PAHs and HCAs. Barlow et al. (24) stated that MOE is the ratio of benchmark dose (BMD) at a lower end of a one-sided 95% confidence interval with a 10% incremental risk (BMDL_10_) supporting the estimated dose. In general, a MOE of 10,000 or higher would be of low concern from a public health point of view if it is based on BMDL_10_ from an animal study and if the overall uncertainties in the interpretation are taken into account (25). A greater number of MOEs represent a lesser probability of causing risk from exposure to a compound (23).

BMD modelling is the state of the science for determining the point of departure for risk assessment. The modelling accounts for all of the data for a particular effect from a particular experiment, increased consistency and better accounting for statistical uncertainties (26). The European Food Safety Authority (EFSA) Scientific Committee (2012) reiterated that an effective and practical method to assess the risk of genotoxic and carcinogenic substances is by MOE and agreed that BMD acts as a better practice that signifies the point of departure in the observable dose–response range (24). BMD is the dose that signifies a low but calculable response, with a lower confidence limit of 95%, which is identified as BMDL (27).

Studies on human exposure to PAHs and HCAs in meat and fish-based products are limited. Most of existing literature has mainly reported limited studies of PAHs or HCAs and human exposure separately (12, 20, 21). This establishes a knowledge gap due to the insufficient details that reported human exposure to both PAHs and HCAs in meat and fish-based products that are widely consumed by the Malaysian population. Therefore, this research was conducted to model the BMDL_10_ of PAH and HCA using BMD software and to calculate the MOEs of PAHs and HCAs by utilising the modelled BMDL_10_.

## Methods

Preferred Reporting Items for Systematic Reviews and Meta-Analyses (PRISMA) was used to aid the collection of relevant articles. PRISMA is a systematic review and meta-analysis that contains 27 checklists and four-phase flow diagrams, which assist an author in making a better report. This method was carried out by reviewing reports where data from various studies were extracted to attain the aim of this study: to calculate the MOEs of PAHs and HCAs by utilising the BMDL_10_ data. The executed systematic review was specified in animal studies on the toxicity of PAHs and HCAs in meat and fish-based products that include the amount of the chemical used, type of animals used, number of animals used, duration of the study and type of cell study. Studies involving humans were excluded as there was no sufficient amount of data reported.

**Figure 1:**
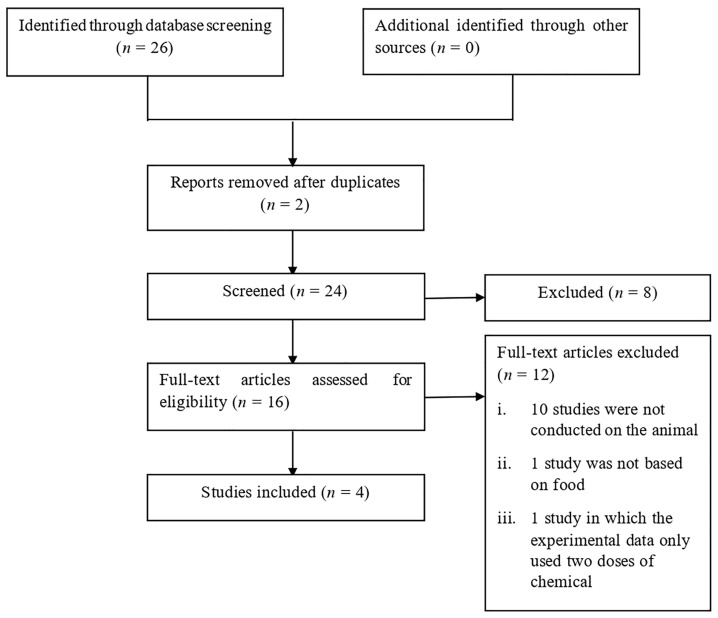
Study selection for inclusion in systematic review

Eight electronic databases were used (Google Scholar, ScienceDirect, Research Gate, BMC Cancer, Taylor & Francis Online, Oxford Academic, Springer Link and Wiley Online Library) to search for previous studies from September 1986 to March 2019 using the following search terms: (Margin of Exposure OR MOE) AND (rats OR mouse OR mice OR animal) AND (chicken OR poultry OR meat OR fish OR Polycyclic Aromatic Hydrocarbons OR PAHs OR Heterocyclic Aromatic Amines OR HCAs). Broad search terms were used to avoid overlooking in any publications. The search was restricted only to the English language. After the first screening, the articles were assessed for acceptance and a few were excluded because of the following reasons: i) the studies were not conducted on animals, ii) the studies were not based on food and iii) the experimental data only used two doses of chemical (Figure 1). A sheet containing extracted data was revised to ensure that all essential information was included and sufficient. The extracted data were then used to model BMDL_10_ using BMD Software version 3.1.2 (https://www.epa.gov/). BMD modelling displayed data of BMD_10_, BMDL_10_, Akaike information criterion (AIC), BMD software recommendation and BMD software notes that were used in calculating MOE. Data on the lowest and highest BMDL_10_ values from each compound were extracted.

The MOE was calculated by dividing BMDL_10_ by the estimated dietary intake (EDI) of the food for human consumption. The present study used the EDI of the sum of three PAHs and the six predominant HCAs in meat and fish-based products reported by Jahurul et al. (20) and Jahurul et al. (21), respectively. These studies reported the dietary exposure of PAHs and HCAs in meat and fish-based products among the adult Malaysian population.

## Results

In the present study, PRISMA was used to collect articles that reported studies on the toxicity of PAHs and HCAs in animals from 1993 to 2013. A total of four published journals were selected (28–31). Subsequently, BMD software was used to model the BMDL_10_ of four different compounds, i.e. FA, benzo[a]pyrene (BaP), 2-amino-3,8,dimethylimidazo[4,5-f]quinoxaline (MeIQx) and 2-amino-1-methyl-6-phenylimidazo[4,5,6]pyridine (PhIP). Table 1 shows the BMDL_10_ value of the recommended model, P-value and AIC of different types of PAHs and HCAs of different genders, duration of administration and target organs. The BMD analysis was conducted using default settings based on the assumption of equal potency of the selected PAHs and HCAs. The results showed that the BMDL_10_ of BaP (2.90 mg/kg bw/day) in females with liver as the target organ was the highest. As for PhIP, the BMDL_10_ of PhIP in males with colon as the target organ was the highest (1.40 mg/kg bw/day).

The highest and lowest modelled BMDL_10_ values were used to calculate a range of MOEs. The BMDL_10_ values on each compound are tabulated in Table 2 based on gender, duration and target organ. The BMDL_10_ of a male administrated with FA for 9 months that had been recognised to have lung adenoma had the lowest BMDL_10_, which was 0.01 mg/kg bw/day. In comparison, the highest BMDL_10_ value (2.90 mg/kg bw/day) was found in a female with colon cancer administrated with PhIP. FA had only been detected to cause tumourigenicity in the lungs.

Table 3 shows the type of compound and the estimated daily intake (mg/kg bw/day) of each compound through processed and cooked meat and fish-based products. The EDI was adapted from a published journal (20, 21) that includes a list of Malaysian dishes. BaP had the lowest EDI whereas PhIP had the highest, which were 0.000040450 mg/kg bw/day and 0.000201836 mg/kg bw/day, respectively.

Table 4 shows the MOEs of two PAH compounds and two HCA compounds. The MOE of FA on both lung adenoma and lung adenocarcinoma was the lowest, which ranged from 19 to 1,412 and the MOE of BaP was the highest, ranging from 41,895 to 71,801. Researchers suggested that a MOE value lower than 10,000 indicates a high concern regarding causing adverse health effects (23). Hence, as shown in Table 4, FA, MeIQx and PhIP had a high concern regarding causing toxicity whereas BaP had a low concern. The MOE of FA on lung adenoma was much lower than that on lung adenocarcinoma.

## Discussion

This study assessed the risk of PAHs and HCAs in meat and fish-based products marketed in Malaysia using the MOE approach and utilising the calculated BMDL_10_ values and estimated daily intake of meat and fish-based products from published data. The calculated BMDL_10_ value of BaP (2.90 mg/kg bw/day) in females with liver as the target organ was the highest. In addition, the highest BMDL_10_ of PhIP (1.40 mg/kg bw/day) was determined in males with colon as the target organ. The BMD software provided the *P*-value and AIC of the BMDL_10_ of the recommended model. Haber et al. (32) stated that besides the best fit of the *P*-value, AIC was also applied to differentiate the model outputs from the dataset as an additional guideline in considering the best fit. US EPA also explained the two-step model selection process. The goodness of fit *P*-value, visual fit and scaled residuals were the criteria that primarily need to be considered in identifying the accepted models. In addition, the model was selected among the acceptable fit left. If the estimated BMDL from each model was undoubtedly close, then the one with the lowest AIC could be used (32). However, Haber et al. (32) also stated that the recommended value of AIC had not been reported or suggested. Hence, other measures were considered, instead of choosing only based on AIC, such as *P*-value, scaled residuals, the visual fit and the evaluation of model influence. At the same time, there were a few sets of data that could not be modelled. The BMD cannot be computed on account of the inconsistency of raw data from a published paper. Haber et al. (32) claimed that a few older research papers might only address observed effects, excluding the incidence data. Some studies might state the mean values and not the overall sets of data that are required to model continuous data. Although these data were not relevant to use, perhaps there were no other choices that could be used to carry out the risk assessment (32).

The BaP BMDL_10_ values that ranged from 1.69 mg/kg bw/day to 2.90 mg/kg bw/day were among the highest. Benford et al. (23) also reported that BMD and BMDL modelling shows higher values on BaP from data of in vivo studies. The BMDL_10_ values in females were much higher than those in males in most types of diseases, except for mammary cancer. Majek et al. (33) reported that the differences between males and females could be affected by sex hormones. Also reported that colon cancer is one of the illnesses strongly affected by gender, with the incidence rates of males being higher than females (34).

BaP had the lowest EDI at 0.000040450 mg/kg bw/day, whereas PhIP had the highest at 0.000201836 mg/kg bw/day. Oz et al. (35) claimed that MeIQx and PhIP are the most common HCAs produced in meat products. In addition, the quantity and frequency of the food consumed might affect human exposure to HCAs (35). BaP was shown to induce stomach and liver cancer (36). In 2006, FAO/WHO claimed that BaP causes tumours of the gastrointestinal tract, liver, lungs, mammary glands and other tissues. Even though BaP has been known to be a carcinogenic PAH marker in food, this compound often cannot be identified in food (16). This might be because each PAH has different metabolism steps or a mixture of PAHs may alter the pathways and their target organ (23).

FA, MeIQx and PhIP had a high concern regarding causing toxicity because their MOE values were less than 10,000. The MOE values of PAHs (i.e. FA) that were less than 10,000 are consistent with those from different researchers (37) who conducted a risk assessment of PAH4 in grilled meat and fish in Turkey (37), Baltic states (38) and Denmark (39). However, the MOE values of HCAs (i.e. MeIQx and PhIP) that were less than 10,000 are in contrast with those from Lee et al. (40), who reported that the MOE of PhIP based on a Korean total diet study was 2,349,000. Manan et al. (41) stated that the difference in exposure may be caused by variations in meal patterns, economic growth, culture, lifestyle and eating habits. Moreover, Pouzou et al. (42) performed a probabilistic assessment of dietary exposure to heterocyclic amines and PAHs from the consumption of meats and bread in the United States but did not calculate the MOE values.

The MOE value of MeIQx in liver cancer ranged from 6,322 to 7,652 which indicates high concern regarding causing toxicity in humans. Zimmerli et al. (43) claimed that the mutagenicity of MeIQx in the liver was related to the large dose range. The researchers (43) also stated that the liver is the primary target organ of most HCA compounds, except for PhIP and MeIQ. In Table 4, PhIP, with the lowest value, was for mammary cancer. It was supported by Zimmerli et al. (43), who stated that the PhIP compound causes tumours in the mammary glands of female rats and causes prostate carcinomas in male rats.

## Conclusion

In the present study, the MOEs of PAHs and HCAs in meat and fish-based products consumed by the adult Malaysian population were calculated by utilising the modelled BMDL_10_ and EDI. The MOE of BaP was higher than 10,000, which indicates that BaP can be considered a low concern. FA, MeIQx and PhIP had MOEs lower than 10,000, which show that these three compounds are of high concern and are a priority for risk management actions.

## Figures and Tables

**Table 1:** Results from a BMD analysis of the data for different organs exposed to different type of PAHs and HCAs using BMD software version 3.1.2, a BMD of 10% and default settings based on assumption of equal potency of the selected PAHs and HCAs

Chemical	Gender	Duration	Type of disease	Model	BMDL_10_ (unit)	*P*-value	AIC
Fluoranthene (FA)	M^a^	6 months	Lung adenoma	Logistic	0.175	0.46	47.03
	F^a^			Gamma	0.17	0.70	48.25
	M^a^	9 months		Dichotomous Hill	0.00	65535	68.08
	F^a^			–	–	–	–
	M^a^	6 months	Lung adenocarcinoma	Gamma	0.21	0.55	25.20
	F^a^			Weibull	0.52	1.00	2.00
	M^a^	9 months		Logistic	0.20	0.84	27.01
	F^a^			Logistic	0.29	0.99	15.77

Benzo[a]pyrene (BaP)	M^b^	104 weeks	Stomach cancer	–	–	–	–
	F^b^			Quantal linear	2.68	0.99	147.33
	M^b^		Liver cancer	Log-logistic	2.36	0.51	127.85
	F^b^			Multistage degree 3	2.90	0.99	92.25

2-amino-3,8,dimethylimidazo[4,5-f]quinoxaline (MeIQx)	M^c^	56 weeks	Liver cancer	Weibull	1.12	1.00	50.32

2-amino-1-methyl-6-phenylimidazo[4,5,6]pyridine (PhIP)	M^d^	104 weeks	Mammary cancer	–	–	–	–
	F^d^			Logistic	0.77	0.75	124.42
	M^d^		Mammary cancer	Quantal linear	0.48	0.98	94.59
	F^d^			Quantal linear	1.28	0.30	73.80
	M^d^		Colon cancer	Weibull	1.40	1.00	43.05
	F^d^			Dichotomous hill	0.82	0.99	27.56

Notes: M = male; F = female;

a Wang & Busby (30);

b Aaslyng et al. (16);

c Kushida et al. (29);

d Carthew et al. (28)

**Table 2:** The highest and lowest value of BMDL_10_ (mg/kg bw/day) of different types of PAHs and HCAs on different genders at different durations of exposure

Chemical		Fluoranthene				Benzo[a]pyrene		MeIQx	PhIP	
Gender		M^a^	F^a^	M^a^	F^a^	M^b^	F^b^	M^c^	M^d^	F^d^
		
Duration		6 months		9 months		104 weeks		56 weeks	104 weeks	
Lung adenoma	Highest BMDL_10_	0.17	0.22	0.05	-	-	-	-	-	-
	Lowest BMDL_10_	0.08	0.09	0.01	-	-	-	-	-	-

Lung adenocarcinoma	Highest BMDL_10_	0.30	0.52	0.19	0.29	-	-	-	-	-
	Lowest BMDL_10_	0.16	0.52	0.12	0.25	-	-	-	-	-

Stomach cancer	Highest BMDL_10_	-	-	-	-	-	2.68	-	-	-
	Lowest BMDL_10_	-	-	-	-	-	1.69	-	-	-

Liver cancer	Highest BMDL_10_	-	-	-	-	2.42	2.90	1.33	-	-
	Lowest BMDL_10_	-	-	-	-	2.03	2.56	1.09	-	-

Mammary cancer	Highest BMDL_10_	-	-	-	-	-	-		-	0.77
	Lowest BMDL_10_	-	-	-	-	-	-	-	-	0.55

Leukemia	Highest BMDL_10_	-	-	-	-	-	-	-	0.95	2.01
	Lowest BMDL_10_	-	-	-	-	-	-	-	0.48	1.28

Colon cancer	Highest BMDL_10_	-	-	-	-	-	-	-	1.91	2.90
	Lowest BMDL_10_	-	-	-	-	-	-	-	0.96	0.82

Notes: M = male; F = female;

a Wang & Busby (30);

b Aaslyng et al. (16);

c Kushida et al. (29);

d Carthew et al. (28)

**Table 3:** The daily intake (mg/kg bw/day) of PAHs (FA and BaP) and HCAs (MeIQx and PhIP) through processed and cooked meat and fish-based products

Chemical		Daily intake (mg/kg bw/day)
PAHs	Fluoranthene	0.000365330
	Benzo[a]pyrene	0.000040450
HCAs	MeIQx	0.000173406
	PhIP	0.000201836

**Table 4:** MOE of PAHs (FA and BaP) and HCAs (MeIQx and PhIP) based on their daily intake in processed and cooked meat and fish-based products, and the highest and lowest BMDL_10_

Type of disease	Chemical			

	Fluoranthene	Benzo[a]pyrene	MeIQx	PhIP
Lung adenoma	19*–614*	-	-	-
Lung adenocarcinoma	328*–1412*	-	-	-
Stomach cancer	-	41895–66185	-	-
Liver cancer	-	50118–71801	6322*–7652*	-
Mammary cancer	-	-	-	2711*–3797*
Leukemia	-	-	-	2362*–9969*
Colon cancer	-	-	-	4045*–14390

Note:

* The MOE that is lower than 10,000 indicate its possible toxicity towards human health
